# Rev. Albert Carrier Bunn, M. D.

**Published:** 1913-02

**Authors:** 


Rev. Albert Carrier Bunn, M. D., Buffalo, 1867, late Rector of St. Matthew's at Brooklyn Manor, Borough of Queens, died December 24 at Ashville, N. C. He was educated at Hobart College and was ordained to the priesthood of the Episcopal Church in 1882. He served as a medical missionary at Wuchang, China, 1874-9, and established St. Peter's and the Elizabeth Bunn Hospitals.

